# Supplementary material for: A set of multi-entry identification keys to African frugivorous flies (Diptera, Tephritidae)
Source: Zookeys. 2014 Jul 24;(428):97–108. doi: 10.3897/zookeys.428.7366 (PMC4143993; doi:10.3897/zookeys.428.7366)
Supplement: Supplementary material 4 — Key to Capparimyia [file zookeys-428-097-s004.zip › SF4_ZooKeys_key to Capparimyia/lucid_player/help/help/setting_up_the_player.htm]

Configuring the Player


# Configuring the Player

## Keeping subsets on restart

By default, if you have chosen one or more subsets
during an identification and you click the *Restart*
button or choose *Restart* from
the *Key* menu, the subsets will
be retained when the key is restarted. If instead you would like any subsets
to be reset (so the key is restarted using all features and entities),
uncheck the *Keep Subsets on Restart*
option on the *Key* menu.

## Allowing misinterpretations

In Lucid keys, some entities may be scored as having a feature *by misinterpretation*. For example,
some groups of wasps are distinctive in having a strikingly narrowed section
or �waist� between the thorax and abdomen, while in other groups of wasps
and in most other insects there is no such narrowing. However, some members
of the �waisted� wasps, such as the common honeybee, have a very short
waist that is almost hidden by the other body segments and is easily overlooked.

If a key to wasps has a feature *Waist
present/absent*, there is a danger that the waist on a honeybee
would be overlooked by a user who would incorrectly describe their specimen
as not having a waste. This could result in a misidentification.

To prevent such errors, the key�s builder will score the honeybee as
*Waist present* (true) and *Waist absent* (*by
misinterpretation*). The Lucid Player treats the by misinterpretation
score as though it were a true score, so the honeybee will be retained
in *Entities Remaining* even if
the waist is overlooked.

However, another user - who is more experienced in identification -
may wish to have such misinterpretations ignored and to only see a list
of all wasps that truly lack a waist. In this case, the honeybee should
be removed from *Entities Remaining*.

The Lucid Player allows users to select these different modes of operation
through the *Allow Misinterpretations*
item of the *Key* menu. With *Allow Misinterpretations* checked (the
default behaviour), entities scored for a state using the by misinterpretation
score will remain in *Entities Remaining*
when the state is selected; with *Allow
Misinterpretations* turned off, such entities will be discarded.

## Retaining uncertain entities

In Lucid keys, some entities may be scored as *Uncertain*
for some states. For example, a key to plants may include features concerning
fruits, but some rare species may never have been observed at the fruiting
stage when the key was being built. In this case the builder of the key
would score fruit features and states for such entities as *Uncertain*.

In normal identifications, it is important for the Lucid Player to treat
entities scored as *uncertain*
for a state in the same way as entities scored *present*
for the state. Using the example above, a user of the key may have a specimen
of the rare species that does have fruits, and hence may be able to address
the fruit features. In such a case, the species should remain in *Entities Remaining* even though the
key builder could not properly score the fruit features.

However, another user may wish to have such uncertain scores ignored
and to only see a list of all species that are *known*
to have fruits of a particular shape or colour. In this case, the species
with unknown fruits should be removed from *Entities
Remaining.*

The Lucid Player allows users to select these different modes of operation
through the *Retain Uncertains*
item of the *Key* menu. With *Retain Uncertains* checked (the default
behaviour), entities scored for a state using the uncertain score will
remain in *Entities Remaining*
when the state is selected; with *Retain
Uncertains* turned off, such entities will be discarded.

## Setting the Best mode

Lucid3 has two modes of the *Best*
function (for more information on Best, see the topic Finding
the best feature � the Best functions). *Find
Best* will search for and open the best feature to address next,
while leaving the features list in its original order, while *Sort
Best* will reorder the features list from best to worst.

To set which *Best* mode to use,
choose either *Find Best* or *Sort Best* from the *Best
Mode* option of the *Features*
menu.

Note that *Sort Best* requires
that the Player is in *List
View*.

## The Lucid3 Matching Methods

Lucid3 matches entities against selected features to determine which
entities match and which do not.

Two options, accessed through the *Match
Method* option of the *Key*
menu, allow you to vary the way the Lucid3 Player handles the matching.

Using the *Any State* matching
method, entities will be kept in *Entities
Remaining* if they match any chosen state of a feature. Using the
*All States* matching method, entities
will only be kept in *Entities Remaining*
if they match all chosen states of a feature.

To understand these modes, consider a feature *Flower
colour* with the following states:

Flower colour  
white  
red  
blue  
yellow

Consider three entities *e1*,
*e2* and *e3*
that have been scored by the key�s builder as follows: *e1*
has been scored for *blue* only,
*e2* has been scored for *yellow* only, while *e3*
has been scored for both *blue and yellow*.

Using the *Any State* match method
in the Player and choosing the state *blue*,
*e1* and *e3*
will remain in *Entities Remaining*
(since both are scored as having blue flowers) while *e2*
will be moved to *Entities Discarded*.
If you choose instead the state yellow, *e2*
and *e3* will remain in *Entities
Remaining* while *e1* will
be moved to *Entities Discarded*.
Finally, if you choose both states blue and yellow, all three entities
will remain in *Entities Remaining*
(that is, the Player is returning all entities scored for either blue
or yellow)

By contrast, using the *All States*
match method and choosing the state blue, only *e1*
will remain in *Entities Remaining*
(since only it is scored for only blue) and *e2*
and *e3* will be moved to *Entities Discarded*.  If
you choose instead the state yellow, only *e2*
will remain in *Entities Remaining*
while e1 and e3 will be moved to *Entities
Discarded*. Finally, if you choose both states blue and yellow,
only *e3* will remain in *Entities Remaining* while *e1*
and *e2* will be moved to *Entities Discarded* (that is, the Player
is returning only entities scored for both blue *and
yellow*).

The normal matching method for most identifications is *Any
State*, as this allows more flexibility between the scores given
by the key�s builder and the answers provided during the identification.
In some circumstances, you may wish to change the mode to *All
States*. This can be done at any time. You should be careful to
understand how this method works before changing to it.

**Note** also that some
authors may set All States as the default Match Method and have scored
their keys, such as diagnostic keys, on this basis.  In
this case, changing the Match Method will cause the key to operate in
a very different way from that intended by the author.

## The Lucid3 Sorting Methods

Lucid3 matches entities against each selected feature to determine which
entities match and which do not.

Two options, accessed through the *Sorting
Method* option of the *Entities*
menu, allow you to vary the way the Lucid3 Player reports the results
of the matching.

In a *Filtered* sort, entities
that do and do not match the selected features are sorted into two lists,
*Entities Remaining* and *Entities Discarded* respectively.

In a *Ranked* sort, all entities
are listed in the *Entities Remaining*
panel (and the *Entities Discarded*
panel is hidden). Entities are prefixed by their percentage match to the
selected features (when using the *Any
State* matching method) or selected states (when using the *All States* matching method). (For more
information on the matching methods, see the topic Lucid3 Matching methods
above)

The entities in a *Ranked* sort
are ranked by their percentage match value. For example, if an entity
matches all the selected features it will have a match value of 100% and
will appear in the top rank of the *Entities*
list. If another entity matches half the selected features (or states)
it will have a match value of 50% and will be ranked lower in the list.

Entities sorted using the Filter Method

Entities sorted using the Ranked Method

## Automating Best and Prune

The *Best* and *Prune
Redundants* functions by default are only applied when you click
the appropriate button or menu item.

However, you may automate these functions, so they are applied whenever
a state is chosen. With these automated, after addressing each feature
the *Features Available* list will
be automatically pruned of redundant features and reordered to show the
next best feature.

To set *Best* and *Prune
Redundants* to be automatically applied, check the menu items *Auto Best* and *Auto
Prune* from the *Automate*
item of the *Features* menu.

If you need to choose several states from one feature before applying
the *Best* and *Prune
functions*, hold down the *Control*
key while choosing the states. The automated functions will be paused
until the control key is released.

## Internationalisation

The *Language* option in the
*View* menu opens a dialog allowing
you to select a display language from any available language files bundled
with the key. Selecting an alternate language will update the user interface
with the translated strings contained within the language file.

## Fonts

The *Font* option, located in
the *View* menu, allows you to
select an alternative font for the user interface. Certain language files,
such as Eastern Asian languages, require extended characters to display
correctly. It is important to select a suitable font when using these
languages.

The *Font Size* option, also
located in the *View* menu, allows
you to increase or decrease the font size for the trees.
